# Pure and Modified Co-Poly(amide-12-b-ethylene oxide) Membranes for Gas Separation Studied by Molecular Investigations

**DOI:** 10.3390/membranes2030346

**Published:** 2012-06-28

**Authors:** Luana De Lorenzo, Elena Tocci, Annarosa Gugliuzza, Enrico Drioli

**Affiliations:** Institute on Membrane Technology ITM-CNR, University of Calabria, 87030 Rende (CS), Italy; Email: ldelore@unical.it (L.L.); e.drioli@itm.cnr.it (E.D.)

**Keywords:** PEBAX, membrane, gas separation, molecular dynamics simulations

## Abstract

This paper deals with a theoretical investigation of gas transport properties in a pure and modified PEBAX block copolymer membrane with *N*-ethyl-o/p-toluene sulfonamide (KET) as additive molecules. Molecular dynamics simulations using COMPASS force field, Gusev-Suter Transition State Theory (TST) and Monte Carlo methods were used. Bulk models of PEBAX and PEBAX/KET in different copolymer/additive compositions were assembled and analyzed to evaluate gas permeability and morphology to characterize structure-performance relationships.

## 1. Introduction

**Scheme 1:**
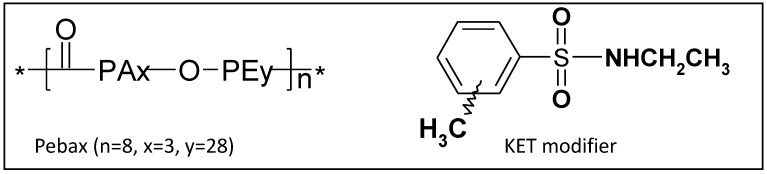
Molecular Structures of PEBAX chain repeat unit and *N*-ethyl-o/p-toluene sulfonamide (KET) modifier.

Today membrane technology has become much more competitive in separation techniques than traditional methods, such as adsorption, cryogenic separations, distillation, *etc.*, since it achieves improved performance at lower cost together with increased energy efficiency and lower environmental impact [1]. The successful development of highly permeable and selective membranes makes a membrane-based process a viable alternative for carbon dioxide capture and for storage of flue gas. However, there is a key technical challenge to be met: That of achieving higher permeability and higher selectivity [2]. Special attention has been paid to the use of membranes in a solubility-selective mode in recent years, obtaining preferential permeation through the membranes of more soluble gaseous species [3,4]. Polymers containing poly(ethylene oxide) units have an interesting CO_2_/N_2_ selectivity. In fact, the copolymer of the PEBAX series, formed by a rigid-semicrystalline block of polyamide (Nylon12, PA-12) covalently linked to amorphous and rubbery co-monomer poly-tetramethylene oxide (PTMO), as indicated in Scheme 1, is very close to the upper bound [2] owing to its high selectivity for polar/non-polar gases. PEBAX-based polymers have been studied extensively in the past decade for membrane-based gas and vapor-separation uses [5,6,7]. A successful strategy for improving CO_2_ and water vapor permeability is the addition of selective polymeric additives to this matrix polymer. Adding organic molecules with different chemical structures to a polymer matrix generally implies a modification of the system morphology, the chemical composition and even the physico-chemical properties change, influencing the transport through the membranes [8,9,10].

Previously, the effects of the addition of *N*-ethyl-o/p-toluene sulfonamide (KET) molecules on the gas permeation properties were investigated [11,12,13,14,15,16,17]. A clear trend of the influence of additives on gas permeability was observed: The gas transport properties of PEBAX improved with increasing additive concentration up to a certain value. On the basis of previous studies, interactions between KET fillers and polymer chains (Scheme 1) were studied resulting in the identification of the effective distribution of molecular compounds at a nanometric level. Also, interactions between the PEBAX matrix and chemical additives KET were investigated at various concentrations in the PEBAX/KET membranes. How modifications in the structure at fully atomistic level affect the resulting transport properties of the membranes is the subject of this paper. Modeling of transport of individual penetrant molecules provided a deeper understanding of the correlations between transport and structural features of the polymeric membrane materials [18,19,20,21,22,23]. Based on a given chemical architecture, a novel synthetic polymer can theoretically be developed and scrutinized for its utility as a separation medium. This concept offers enormous potential for development in the material sciences. This fact, coupled with increasing computational power and efficiency, allows macromolecular design to be brought to a wider range of users. However, there are still some challenges that must be overcome in the use of molecular simulation as a tool for membrane design, e.g., accurate and robust force field development, and the preparation of “correct” packing models, are challenges currently being addressed. As a contribution to this end, grand canonical Monte Carlo (GCMC) and molecular dynamics (MD) simulations are undertaken in this investigation, in conjunction with the COMPASS force field, to provide estimates of the transport properties of five polyatomic gases (H_2_, N_2_, CO_2_, O_2_ and CH_4_) and water vapor in PEBAX. The predicted values for solubility and diffusivity are compared with experimental values gathered from previous studies [10,11,12,13] and used to assess the predictive capability of the techniques. Furthermore, several previously proposed solubility and diffusivity correlations are also assessed for their predictive capability. 

## 2. Theoretical Section

Molecular simulations were performed using the Materials Studio (5.0) software of Accelrys, Inc. (San Diego, CA). Amorphous polymer packings were constructed using the Theodorou/Suter method [24,25] as implemented in the Amorphous-Cell module [26]. MD simulations were performed with the Discover software using the COMPASS force field [27,28]. The Materials Studio (5.0) software was run on a supercomputer (CINECA cluster, Bologna, Italy) and on PC hardware.

### 2.1. Preparation of Polymer Models

**Figure 1:**
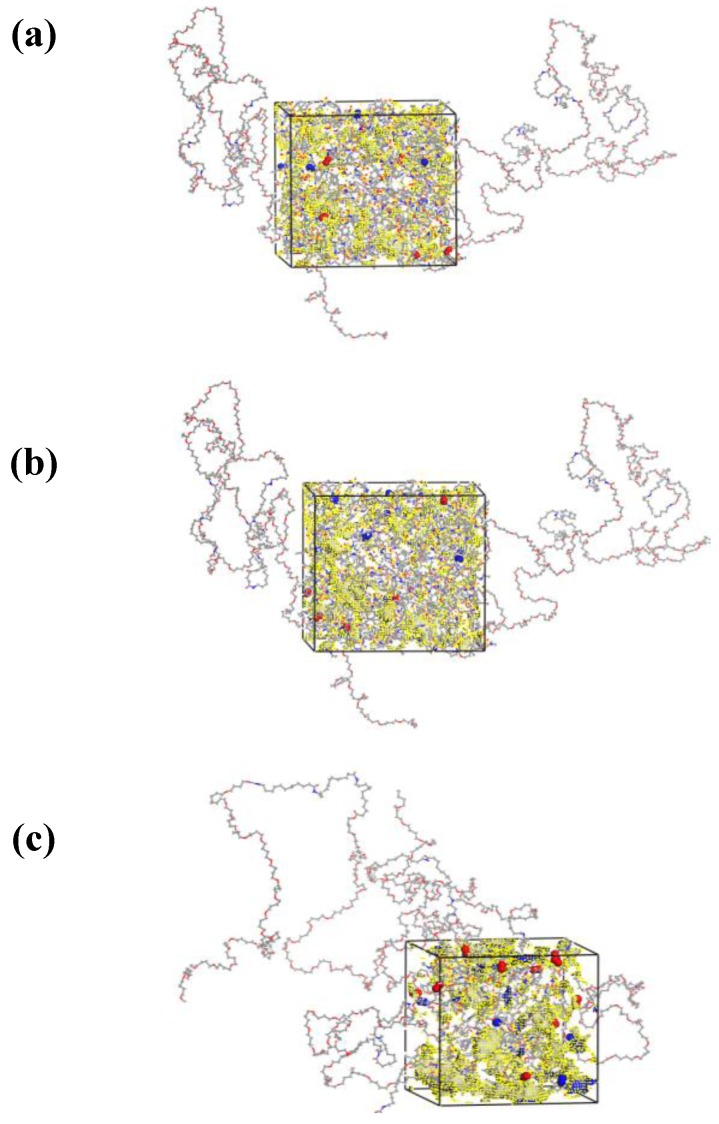
3D Equilibrated simulation models of (**a**) PEBAX/30 wt % KET; (**b**) PEBAX/50 wt % KET; (**c**) PEBAX/70 wt % KET. The region of free volume inside the systems is colored in yellow.

Three-dimensional boxes of PEBAX^® ^2533 were constructed with different KET concentrations (Figure 1).

The applied basic techniques of packing and equilibration are described in detail elsewhere [15]. Single chains of the copolymer PEBAX^® ^2533 were constructed by alternating co-monomers of polyamide-12 (PA-12) and poly-tetramethyleneoxide (PTMO) according to the experimentally observed weight percentages of 20 wt % of PA-12 and 80 wt % of PTMO [29,30]. A co-polymer chain of eight co-monomer units, with 24 units of PA-12 (888 atoms) and 224 units of PTMO (2912 atoms) was used as a template chain for the adjacent initial packing with the Amorphous Cell module. In every packing model each polymer chain was grown, one after the other, under periodic boundary conditions at 308 K and at an initial density of about 75% of the experimental value. This procedure is commonly used to represent bulk amorphous systems and has been proven to be quite accurate in replicating the behavior of experimental polymeric systems [15,31,32]. 

Additionally, every simulation cell contained 600 argon molecules (randomly distributed) as obstacles to avoid ring catenation during the chain growth. KET and water molecules were also introduced in the simulation boxes. The number of molecules of KET, in the racemic ortho/para mixture of 50 wt %, was added to reach the following co-polymer/filler weight compositions: 70/30; 50/50 and 30/70 wt %. The number of water molecules corresponding to the experimentally measured amount of sorbed water in the modified membrane for different PEBAX/KET weight percentages [14] was added in each simulation box. For the chain growing the Theodorou-Suter method was applied, as mentioned above. The Argon molecules were removed in three stages and after each removal, cycles of energy minimization and NVT dynamics (the canonical NVT ensemble is characterized by a constant number of molecules *N*, volume *V* and temperature *T*) were conducted according to the downscaling procedure [20]. After removing the obstacle molecules, the equilibration of the packing models was carried out in the following sequence of simulation steps: (i) 50 ps NVT-MD simulations at 600 K (a simulated annealing); (ii) 20 ps NVT-MD simulations at 308 K (back to target temperature); (iii) 20 ps NPT-MD at 308 K and 10 bar with a time step of 0.1 fs (for the first time volume fluctuations are allowed in the system); followed by (iv) 500 ps NPT-MD at 308 K and 1.0 bar with a time step of 1.0 fs; and (v) a long continuative NPT-MD simulation with the same conditions over 7 ns (all). For the system containing water molecules the duration was 5 ns. The final densities of the three packing models are given in Table 1. The density values of the experimental PEBAX/KET membranes are all at 1.0 g/cm^3^. As can be seen by comparison with Table 1 they agree with the experimental density. Figure 1 shows the three final packing models where the amorphous structure of the cubic boxes can be clearly seen.

**Table 1:** Properties of the atomistic packing models for three samples of PEBAX/KET at different compositions. DP is the degree of polymerization.

Model		DP (–)	N Atoms (–)	N KET	Density, *ρ_simul._*(g/mol)	Cell length (Ǻ)
**PEBAX/KET 70/30 + 10 CH_4 _+ 10 CO_2_**	I BOX	8	5102	46	0.8680	39.00
	II BOX	8	5102	46	0.8888	38.69
	III BOX	8	5102	46	0.8883	38.70
**PEBAX/KET 70/30 + 10 H_2 _+ 10 O_2 _+ 10 N_2_**	I BOX	8	5082	46	0.8929	38.64
	II BOX	8	5082	46	0.8925	38.66
	III BOX	8	5082	46	0.8921	38.65
**PEBAX/KET 50/50 + 10 CH_4_ + 10 CO_2_**	I BOX	8	6662	106	0.9412	42.32
	II BOX	8	6662	106	0.9419	42.31
	III BOX	8	6662	106	0.9432	42.29
**PEBAX/KET 50/50 + 10 H_2 _+ 10 O_2 _+ 10 N_2_**	I BOX	8	6642	106	0.9469	42.24
	II BOX	8	6642	106	0.9460	42.26
	III BOX	8	6642	106	0.9472	42.24
**PEBAX/KET 30/70 + 10 CH_4_ + 10 CO_2_**	I BOX	8	10354	248	1.0473	48.34
	II BOX	8	10354	248	1.0465	48.36
	III BOX	8	10354	248	1.0469	48.35
**PEBAX/KET 30/70 + 10 H_2 _ + 10 O_2 _+ 10 N_2_**	I BOX	8	10334	248	1.0483	48.33
	II BOX	8	10334	248	1.0490	48.32
	III BOX	8	10334	248	1.0494	48.32

### 2.2. Calculation of Diffusion Coefficients

In order to enhance the sampling efficiency ten gaseous molecules of the same kind were inserted into each polymer structure and then the polymeric boxes were equilibrated. Diffusion coefficients were calculated from the slope of the plots of the mean square displacements of gases versus time using the Einstein relation:

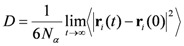
(1)
where 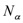 is the number of diffusing molecules of type *α*, 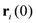 and 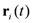 are the initial and final positions of molecules (mass centres of particle *i*) over the time interval *t*, and 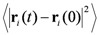 is the mean square displacement (MSD) averaged over the possible ensemble. The Einstein relationship assumes a random-walk motion for the diffusing particles [33].

### 2.3. TST Method

The transition-state theory (TST) is a well-established methodology for the calculation of the kinetics of infrequent events in numerous physical systems [34,35]. These calculations are at the same time a second validation criterion (after the density) for the quality of the developed packing models. With the help of an existing software tool [36] we calculated diffusion coefficients (*D*) of small molecules in polymer matrices, their solubility coefficient (*S*) in the matrix, and the respective permeability (*P* = *D* × *S*). According to the TST method the gas transport mechanism across a dense polymer system is described as a series of activated jumps. For each transition a “reaction trajectory”, leading from a local energy minimum to another through a saddle point in the configuration space, is tracked and the transition rate constant is evaluated. 

First, the free energy to insert a gas molecule in the polymer packing is calculated for all points on a three-dimensional (3-D) grid spacing laid over the packing cell. These data are then used to identify minimum energy insertion sites (“holes”) and to determine transition probabilities from site to site. The penetrant molecules were represented by united atom spheres with the PCFF parameters given in Table 2. 

**Table 2:** Physical properties of six permanent gases used in the experimental and theoretical characterization.

Gas	Critical Temperature (K)^a^	Lennard-Jones Diameters *d_LJ_* (Ǻ)	*ε* (KJ·mol^−1^)
**H_2_**	33.2	2.93	0.307
**O_2_**	154.6	3.46	0.980
**N_2_**	126.2	3.698	0.790
**CO_2_**	304.2	4.00	1.881
**CH_4_**	190.6	3.817	1.231
**H_2_O**	647.1	3.166	0.650

^a^ Critical temperature is a common measure of penetrant condensability. Penetrant solubility in typical polymers increases with increasing penetrant condensability [37].

Gusev and Suter set up the original TST method [34] taking into account the thermal vibrations of the polymer matrix [35,38] with the assumption that the polymer atoms execute uncorrelated harmonic vibrations around their equilibrium positions to accommodate the guest molecules. These assumptions may be considered valid only for the diffusion of small gas molecules such as (He, O_2_, N_2_, CH_4_); it is not appropriate for larger molecules (CO_2_) that may force the polymer atoms in the vicinity of the penetrant to undergo substantial local relaxations to accommodate the guest molecule. Once the corresponding jump probabilities are determined, the trajectory of the penetrant molecules are calculated by a Monte Carlo (MC) type procedure and the diffusivity is extracted from the slope of the mean square displacement (MSD) versus time at long times, when Fickian diffusion is established.

### 2.4. Calculation of Solubility Coefficients

The solubility coefficients, *S*, were obtained from grand canonical Monte Carlo (GCMC) simulations by fitting the sorption isotherm obtained from every simulated box to a straight line through the origin and taking the slope to be the solubility coefficient. In this procedure, a Metropolis [39] algorithm is used to accept or reject an insertion and deletion of a sorbate molecule. 

The probabilities of addition and deletion of a sorbate molecule are given as:
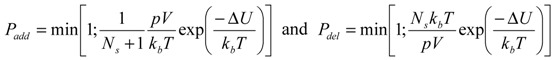
(2)
where *U* is calculated from the sum of non-bonded (*i.e.*, Coulombic and van der Waals interaction) energies, *N_s_* is the number of sorbate molecules. The addition is accepted if the energy change ∆*U*, is negative or if the Boltzmann factor is greater than a random number generated between 0 and 1.

The solubility coefficient *S*, is then obtained from the slope of the sorption isotherm as:

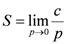
(3)
where *c* is the sorbed gas concentration and *p* is pressure; *S* is expressed in units of (cm^3 ^(STP)/cm^3 ^polymer).

Isotherms were determined for five gases at six pressures over a pressure range from 0.05 atm to 0.3 atm using the SORPTION module [26]. At each pressure, 10^6^ steps of GCMC calculations were performed using an initial equilibration period of 5000 steps. The charge interaction was considered and the non-bond cut-off was set to 12 Å. The GCMC solubility coefficient of each gas at infinite dilution was computed by fitting the sorption isotherm obtained from every simulated box to a straight line through the origin and taking the slope to be the solubility coefficient.

### 2.5. Pair Correlation Functions: (RDF) Analysis

The pair correlations functions *g_AB_(r)* [33] were used for evaluating the relative positions of selected atoms, molecules or chemical groups in our systems at microscopic scale. *g_AB_(r)* represents the probability of finding a pair of particles *AB* at a distance *r (dr)* normalized with respect to the probability expected for a completely random distribution at the same density. 

*g_AB_(r)* are evaluated as:

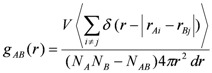
(4)
where *A* and *B* represent two kinds of particles. The system has a volume *V* and contains *N_A _*particles of kind *A* and *N_B_* particles of kind *B* with *N_AB_* particles belonging simultaneously to both kinds. 

Vectors *r_Ai_* and *r_Bj_* represent the position of particle *i* of kind *A* and particle *j* of kind *B*, so that 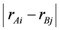 is the distance between those two particles. The term 
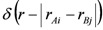
 in Equation 4 is set to unity when 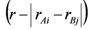 ≤ *dr* (*i.e.*, the difference between the desired and the actual distance among the two particles is smaller than a tolerance factor *dr*) and to zero otherwise.

### 2.6. Atom Volumes & Surfaces

The Atom Volumes & Surfaces of the MS software [26] tool creates a field that contains values of some distance functions, such that isosurfaces of that field characterize the geometry and solvent interaction of an atomistic structure in a variety of ways. Thus, atom volume fields and atom volume surfaces are specialized versions of the equivalent general-purpose volumetric objects. Once these objects have been created using the Atom Volumes & Surfaces tool, the resulting atom volume fields and surfaces are displayed on the Analysis tab of the Atom Volumes & Surfaces dialog. The first volumetric object displayed in the tree view is named the Atom Volumes Field. For the Connolly task, this is a field whose value at each point in space corresponds to the depth in the nearest Connolly probe of a given radius, as it rolls over the van der Waals surface of the atomistic structure [40]. 

## 3. Results and Discussion

### 3.1. Morphological Investigations: Chain Mobility

**Figure 2:**
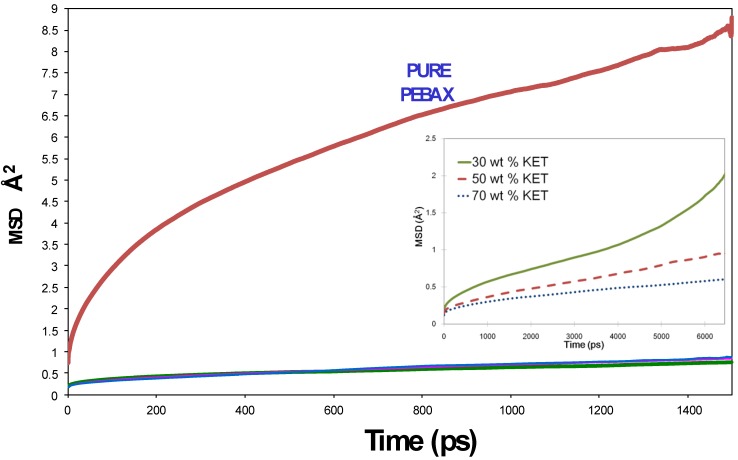
Polymer chain mean squared displacements curves (MSD) in pure PEBAX (time = 1.5 ns) and PEBAX/KET models (time = 7 ns).

In a previous work fully atomistic and experimental investigations of the microstructure in a poly(ether-b-amide) membrane containing the amphiphilic sulphonamide, KET, were performed [17] giving information on the compatibility between KET and PEBAX, and the presence of nanoclusters of KET inside the matrix. At different KET composition modeling, DSC and IR analyses indicated that PEBAX matrixes show some segregation phenomena with subclusters of KET molecules, also at low compositions. How the presence of nanometric-sized KET clusters modifies the transport properties is the aim of this work. In order to investigate the general effect induced by the presence of KET in the polymer structure, we analyzed the motions of the atoms of the polymer chain in the pure and modified PEBAX membranes. The displacements of the polymer chain were calculated by performing dynamic runs on the NPT ensemble on the equilibrated 3D models of pure PEBAX (time of simulation = 1.5 ns) and at different KET compositions (time of simulation = 7 ns); the polymer mean squared displacements curves (MSD) *vs.* time of simulation are shown in Figure 2. 

The MSD curves indicate that the mobility of the atoms of pure PEBAX is greater than that of PEBAX/KET systems, indicating that the presence of KET reduces the mobility of the PEBAX chain. The global effects lead to more rigid and packed structures compared with the pure PEBAX. In PEBAX/KET systems, mainly owing to the bulky aromatic structure and partial double-bond character of the amide linkage of KET molecules, the overall stiffness of the mixed systems gave rise to a reduced mobility of the chains, confirming the reduced rotational freedom of KET observed experimentally with IR spectra [10]. It is also interesting to note that the decrease of chain mobility of 70 wt % KET was examined by DSC [10,13] and by hydrogen bonding analysis, between the sulphonamide moiety and the pseudo-stacked orientation of the aromatic rings of KET [17].

### 3.2. Gas and Vapor Permeability

The reliability of the equilibrated PEBAX/KET models at different additive compositions (30 wt %, 50 wt %, 70 wt %) was also tested by comparing simulated permeability coefficients of five gas molecules, H_2_, O_2_, N_2_, CH_4_ and CO_2_ and water vapor with experimental data already available from Gugliuzza *et al.*, [10,11,12]. Table 3 summarizes the theoretical transport parameters and the available experimental values for PEBAX/KET systems in different compositions. Equilibrated amorphous cells were used in order to estimate the diffusion coefficients using direct molecular dynamics and TST calculations, and the solubility coefficients with Grand Canonical Monte Carlo (GCMC) and TST calculations.

**Figure 3:**
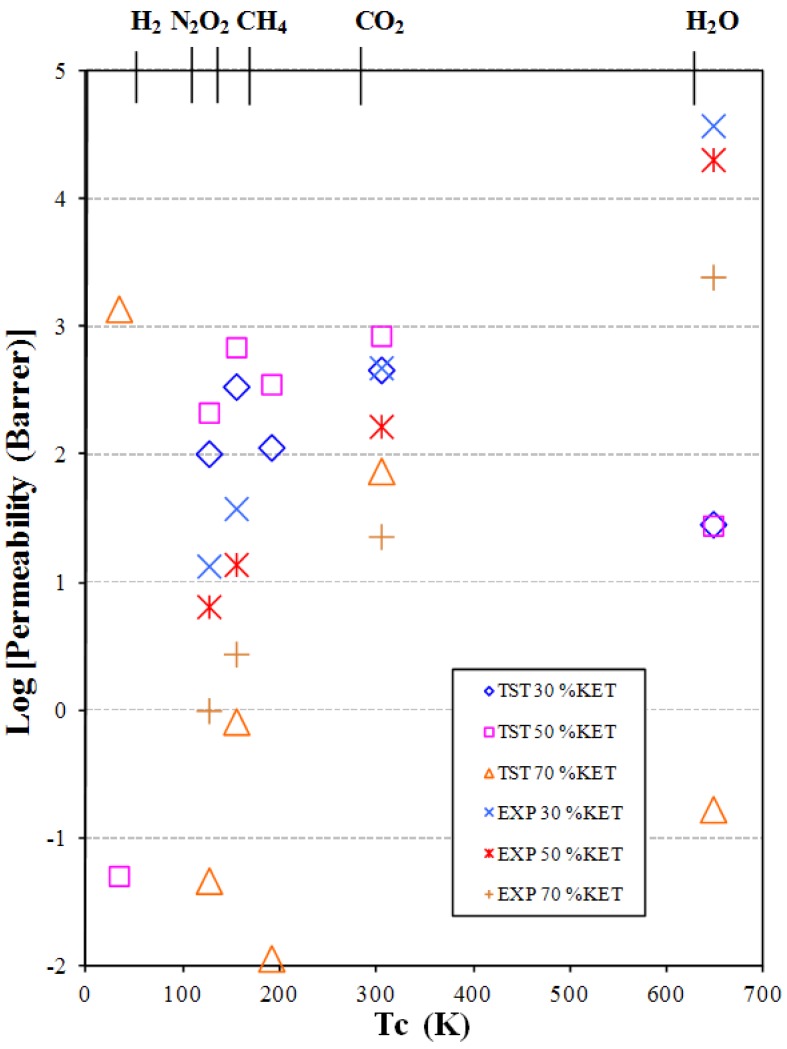
Transition-state theory (TST) calculated and experimental permeability data in modified PEBAX/KET models.

The results of the permeability coefficients [*P*, [cm^3 ^(STP)/(cm^2^·s·cm Hg)] × 10^−10^] *vs.* critical temperatures (*Tc*) are shown in Figure 3.

**Table 3:** Theoretical and experimental data: permeability, diffusion and solubility coefficients of PEBAX/KET membranes.

	Gas	TST Permeability coefficient, *P* barrer ^a^	EXP Permeability coefficient, *P* barrer ^a^	TST Solubility coefficient, *S* (cm^3^_STP_/cm^3^·cm Hg)	GCMC Solubility coefficient, *S* (cm^3^_STP_/cm^3^·cm Hg)	EXP Solubility coefficient, at 25 °C, *S* (cm^3^_STP_/cm^3^·cm Hg)	TST Diffusion coefficient, *D* (cm^2^/s) × 10^−6^	MD Diffusion coefficient, *D* (cm^2^/s) × 10^−6^	EXP Diffusion coefficient, *D* (cm^2^/s) × 10^−6^
**PEBAX/30KET**	**H_2_**	2.35 × 10^−3^	–	4.27 × 10^−3^	1.5 × 10^−3^	–	5.5 × 10^−5^	444.40	–
	**CO_2_**	461.71	480.00	0.43	0.2933	0.047	0.11	3.33 × 10^−4^	1.021
	**O_2_**	342.11	38.09	0.038	2.6 × 10^−3^	–	0.88	0.64	–
	**N_2_**	102.16	13.52	0.026	4.8 × 10^−3^	–	0.38	0.41	–
	**CH_4_**	114.33	–	0.097	0.128	–	0.12	0.017	–
	**H_2_O**	28.70	3.75 × 10^4^	0.57	0.222	7.93	5.09	0.007	0.473
**PEBAX/50KET**	**H_2_**	0.052	–	0.00343	3.42 × 10^−3^	–	1.5 × 10^−4^	364.3	
	**CO_2_**	852.37	167.00	0.5182	0.6115	0.056	0.16	1.16 × 10^−4^	0.298
	**O_2_**	694.08	13.92	0.0428	2.6 × 10^−3^	–	1.62	2.038	–
	**N_2_**	214.21	6.52	0.0300	6.8 × 10^−3^	–	0.713	1.652	–
	**CH_4_**	356.58	–	0.1146	0.1793	–	0.31	3.33 × 10^−3^	–
	**H_2_O**	28.00	2.033 × 10^4^	0.63	0.238	8.90	4.46	0.012	0.228
**PEBAX/70KET**	**H_2_**	1388.16	–	0.00276	0.0021	–	10.55	174.30	–
	**CO_2_**	74.93	23.00	0.2978	0.1878	0.039	0.025	3.3 × 10^−5^	0.059
	**O_2_**	0.824	2.77	0.0306	0.0034	–	2.7 × 10^−3^	0.095	–
	**N_2_**	0.047	1.004	0.0198	0.0074	–	2.3 × 10^−4^	4.5 × 10^−3^	–
	**CH_4_**	0.012	–	0.0830	0.7368	–	1.4 × 10^−5^	1.67 × 10^−4^	–
	**H_2_O**	0.17	2455	0.57	0.294	2.29	2.97	5.00 × 10^−4^	0.107

**^a ^**1 barrer = 10^−10 ^cm^3^_STP _cm/(cm^2^·s·cm Hg)

Two items of information can be obtained:

(1) The permeability increases as the additive concentration decreases.
(2) The permeability increases from small non-polar gases to larger and polar ones.

The first trend, *i.e.*, increasing polymer matrix polarity due to the introduction of KET additives decreases the permeability to all penetrants, could be related to the increased rigidity of the structure, which means a decreased mobility of the chains. 

Generally in the separation of CO_2_ from small gas molecules, if one considers the diffusivity selectivity based on size, the diffusivity of small gases will always be greater because of their smaller kinetic diameter, and therefore higher than that of CO_2_ (Table 2). This tendency is visible for experimental data with membranes with 30% KET showing a higher value than 50% and 70%. The theoretical trend is a little different: Fifty percent KET has the highest value, greater than 30% and 70% for which there is a drastic reduction. The second consideration is validated by the fact that membranes based on PTMO80/PA-12 have high permeation values, as the penetrant species is a gas with high condensability characteristics, that is, a gas with a high critical temperature and high polar/non-polar gas selectivity. For CO_2_ and water the permeability errors of ±10.00 and ±7.4 were estimated, respectively. These indicate the limitation of these types of simulation for condensable gases. The sorption capability of a gas is generally a function of the condensability of the component to be separated. Since CO_2_ is more easily condensed than H_2_, O_2_, N_2_, as evidenced by its much higher critical temperature (Table 2), the solubility of CO_2_ in the polymer tends to be greater than that of small gas molecules. Experimental values of the permeabilities follow this tendency: CO_2_ and water exhibit the highest values. Theoretical data of O_2_, N_2_, CO_2_ follow this trend (but are overestimated for the models with 30 wt %, 50 wt %). Greater deviations are found for H_2_O, H_2_ and CH_4_. H_2_O values are downscaled by some orders of magnitude in comparison with the real ones. H_2_ and CH_4_ are exceptions for the first 70% KET exhibiting the highest value and the second the lowest value of permeability, but no comparison with experimental data for H_2_ and CH_4_.

**Figure 4:**
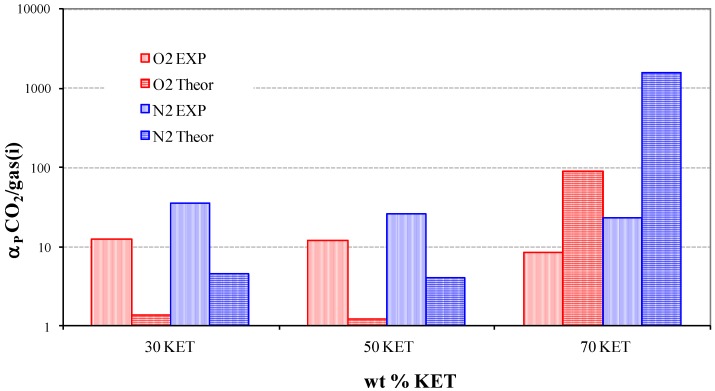
Selective permeability α(CO_2_/N_2_) and α(CO_2_/O_2_) in modified PEBAX/KET systems.

For water we can explain this behavior better, finding relations with our previous paper [17] where we analyzed the effect of KET on the morphology of PEBAX. We know that segregation phenomena have been found and that KET seems to prefer a migration toward polar moieties of the polymer chain, suggesting a progressive saturation of hydrophilic sites of the polymer (*i.e.*, hard polyamide, PA, and soft poly-tetramethyleneoxide, PTMO) with a rising loading of filler. Theoretical selectivity permeability *α*(CO_2_/N_2_) and *α*(CO_2_/O_2_) in modified PEBAX/KET membranes were compared with the experimental data (Figure 4). The experimental *α*(CO_2_/O_2_) is almost constant. Interestingly, the experimental data points of *α*(CO_2_/N_2_), with the exception of 50% KET, are near the upper bound limit of Robeson [2]. The theoretical data are lower than the experimental ones for PEBAX/KET with 30% and 50%. An increase in selectivity is shown when the concentration of KET reaches 70%. In those cases the theoretical values are higher than the experimental ones. Theoretically the selectivity is highest when the KET concentration increases.

### 3.3. Solubility coefficients

Experimental solubility (*S*) is determined by the measured permeability and diffusivity under the assumption that the solution-diffusion mechanism is obeyed (*S* = *P*/*D*) [41]. 

It is known that the addition of KET allows the performance of the traditional polymer “PEBAX” to change, *i.e.*, the water vapor permeability was influenced by the concentration and hydrophilicity of the additives [10]. It is interesting to see what happens in theory.

**Figure 5:**
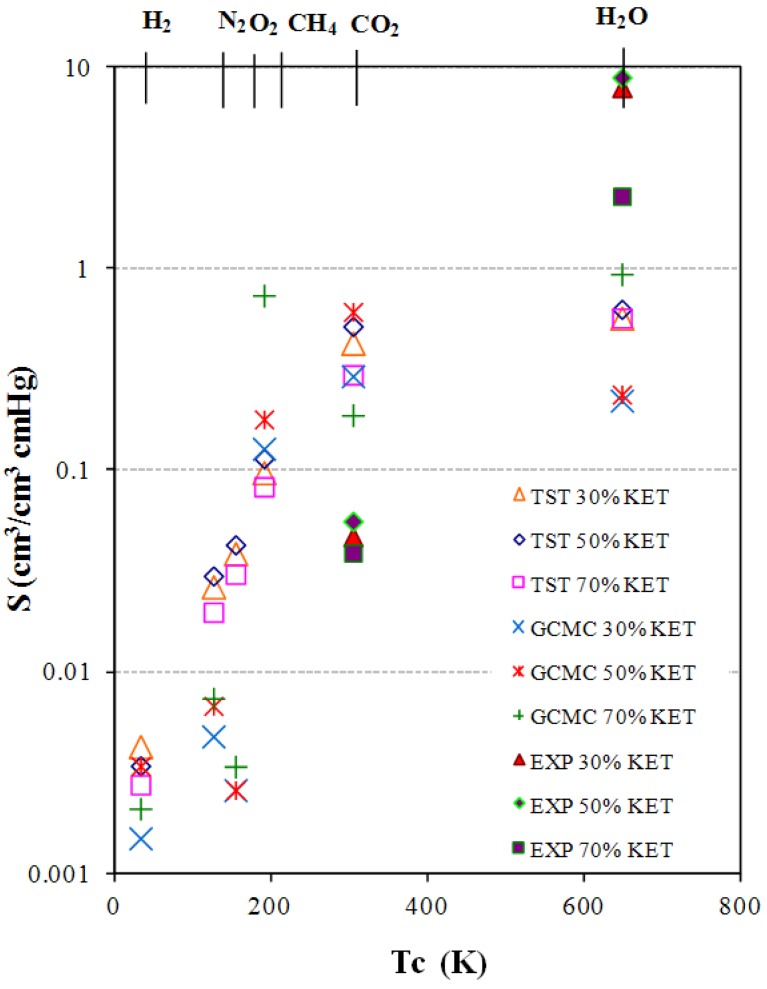
TST and GCMC solubility coefficients *vs.* Critical Temperature (*Tc*) of H_2_, N_2_, O_2_; CH_4_, CO_2_, H_2_O. Experimental data are available and reported only for CO_2 _and H_2_O gases.

Theoretical gas solubility coefficients were obtained by using the TST and the GCMC simulation methods. Errors were found in a range of 0.001 to 0.005. The critical temperature of the probe gas was used as a measure of molecular interaction [37]. From a theoretical standpoint, the critical temperatures of the probe gases studied can be considered to be a measure of van der Waals interaction between molecules. A higher critical temperature corresponds to a greater van der Waals interaction. Solubility increases with increasing condensability of the penetrant species, the penetrants with higher critical temperature being more condensable and, therefore, more soluble, especially in membranes bearing groups with a high degree of polarity (Figure 5).

The opposite behavior was observed with regard to the permeability curves: The higher the amount of KET in the matrix the higher the solubility.

TST data follow this trend: Solubility of PEBAX 50% KET > 30% > 70% for all gases. Instead GCMC values show that solubility of 70% KET > 50% > 30%, *i.e.*, a completely inverted behavior. Exceptions are found for theoretical data on CO_2_: The highest values for both TST and GCMC is that of 30% KET, followed by 50% KET and ending with 70% KET. For carbon dioxide the comparison with experimental data indicates that TST and GCMC are both bigger. This trend is opposed to that of water vapor or the theoretical solubilities (TST and GCMC), which are lower than found in the experimental data.

Also TST data for the small and non-polar gases such as H_2_, O_2_, N_2_ are higher compared with GCMC values for all the concentrations, but methane TST data are lower than GCMC. 

Solubility is basically a thermodynamic property and an overestimation suggests a wrong assumption of an excessively high polymer-penetrant interaction. 

The solubility coefficient of H_2_O obtained from both theoretical methods employed is significantly lower than the effective solubility coefficients measured. This could be explained considering the fact that for both TST and GCMC simulations it is assumed that the polymer matrix is rigid, therefore the free volume elements of the matrix are exclusively used as a sorption site of the membrane. The relatively small dimension of the boxes also does not permit a correct statistic to be reached. The water insertion necessitates a certain dilation of the polymer matrix. However, it is also possible to attribute the discrepancy to the inaccuracy of the interaction potentials, and incorrect use of the Lennard-Jones parameters in the description of a molecule such as water, which is strongly polar. 

These limitations are manifested in the inability to estimate accurately the solute chemical potential when the insertion of large test particles is attempted in free volume elements with a size comparable with the molecular size, a problem referred to as the “insertion problem” by Theodorou [37,42]. From the comparison of experimental and theoretical water solubility coefficients, an acceptable agreement is observed. This occurs in spite of the intrinsic limitations of the TST and GCMC methods in evaluating the water solubility. An increase of water solubility is observed for additive concentrations up to 50 wt %, whereas it decreases for the membranes with 70% on weight of KET.

**Figure 6:**
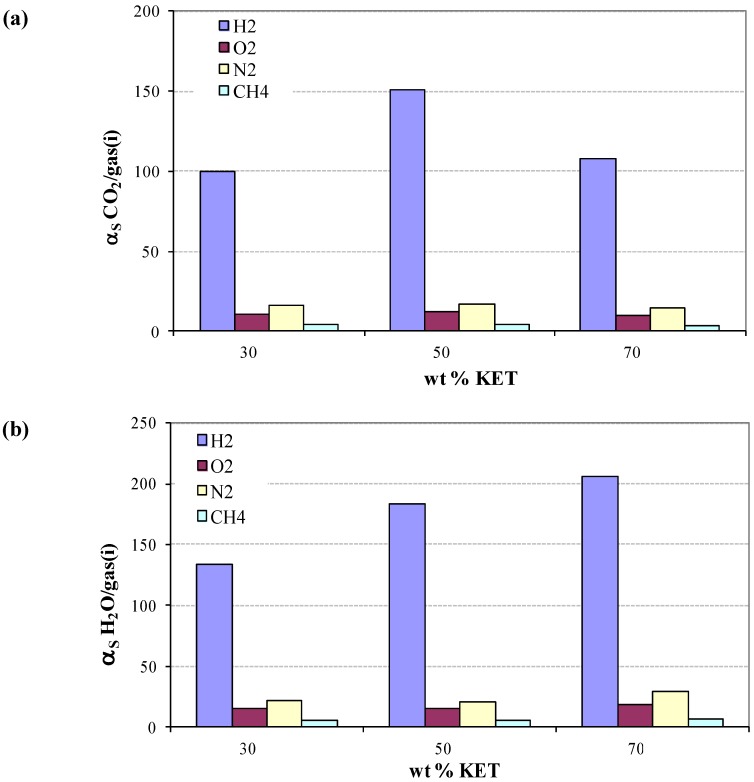
Solubility selectivity of (**a**) CO_2_ and (**b**) H_2_O to non-polar gases at different concentrations of KET.

The theoretical solubility selectivity, calculated via the TST method, of carbon dioxide and water in comparison to non-polar gases show (Figure 6a,b) the same trend in all concentrations: The highest ratio is shown for hydrogen and the lower for methane. 

### 3.4. Diffusivity of Gases and Free Volume in PEBAX/KET Membranes

The self-diffusion coefficients were calculated from the slope of the mean square displacement curves of the MD runs via averaging over all simulated penetrant molecules of a given kind. A simulation time of 5 ns was considered for water and 7 ns for gases. The theoretical values are much lower than the experimental values, also when small dimension gases are simulated (Table 3). This behavior could be ascribed to: (1) Rigidity of the mixed matrix system due to the enlargement of the nano cluster size of KETs; (2) the free volume; (3) the duration of runs. Although it is 5 ns, it is not enough to reach the normal diffusion regime. This could be the major reason for the discrepancy between theoretical and experimental data. Therefore MD runs need more time. Also the TST method gives accurate values for small gas molecules, but for non-spherical polyatomic probes where consideration of penetrant shape is important, results are found to be less comparable. For those reasons the membranes containing 70% KET show worse results.

**Figure 7:**
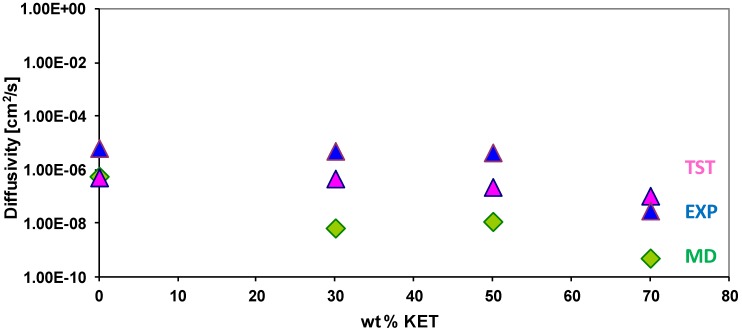
Experimental and theoretical diffusivity of H_2_O in PEBAX membrane with different wt % of KET.

Figure 7 shows the comparison of the theoretical and experimental water diffusivity for varying PEBAX/KET compositions. From the comparison with the experimental data of Gugliuzza *et al.*, [10,13] an acceptable agreement with the calculated diffusion coefficients is observed, taking into account the limitations of the method for calculating the diffusion coefficients. The TST method overestimates the diffusivity of H_2_O, and, on the other hand, MD gives a better reproduction of the experimental water diffusivity than TST only for the pure PEBAX system, because the introduction of an additive like KET with rigid aromatic rings leads to a reduction of the chain mobility, as seen in the previous section. The reason for this is the immobilization of polymer chains due to the hydrogen bond formation or steric hindrance by aromatic structures. The observed effect increases at increasing wt % KET in PEBAX membranes so the value of water diffusivity by MD is undervalued especially for the system with 70 wt % KET.

The results of MD calculations indicate a similar difficulty with polyatomic gases. The lack of accuracy in estimates for diffusivity was previously attributed to the process of potential parameter fitting [43]. It was proposed that the parameters of PCFF force field, the precursor of COMPASS, should be optimized to describe polymer morphology at the expense of accuracy in transport property prediction [43]. This may explain the lack of predictive accuracy. Despite this, these calculations are still within the “factor of three to five” accuracy typically observed in MD simulations [42].

A Connolly surface was created for the equilibrated models of PEBAX/KET at 30 wt %, 50 wt % and 70 wt %, by the atom, volumes and surface module in Materials Studio [26] using a fine grid resolution and a Connolly radius set to 1.82 Å (the kinetic radius of N_2_). The occupied volume (OV), the surface area (SA), the free volume (FV) and the fractional free volume (FFV) for the models at different compositions are listed in Table 4. 

**Table 4:** The occupied volume (OV), the surface area (SA), the free volume (FV) and the fractional free volume (FFV), calculated by Atom Volumes & Surface Tool for the models PEBAX/KET at different compositions of modifiers.

wt % KET	OV (Ǻ^3^)	SA (Ǻ^2^)	FV (Ǻ^3^)	FFV
**30**	50445	11472	8873	0,149
**50**	64826	14003	10972	0,144
**70**	102115	17220	10875	0,096

The occupied volume increases with the higher loading of KET and the same trend can be observed for the fractional free volume that decreases. This effect together with the increased rigidity of the polymer chains owing to the presence of KET molecules can explain the low diffusivity coefficients of gases and water vapor evidenced both at experimental and theoretical levels. 

### 3.5. Structural Analysis: Radial Distribution Functions

The fundamental contribution to the transport of CO_2_ in PEBAX based membranes is given by the solubility, *i.e.*, the interaction between the gas and the polymer matrix. For the pure PEBAX we have already found [15] that the soft block of the co-polymer (PTMO) plays the main role in the solubility of CO_2_. 

In order to explain the high CO_2_ solubility, associations were investigated between CO_2_ and possible sites of interactions of the polymeric chains. Nitrogen atoms of amide groups, in PA-12 and oxygen atoms of PTMO block were explored by the radial distribution functions (RDF). 

**Figure 8:**
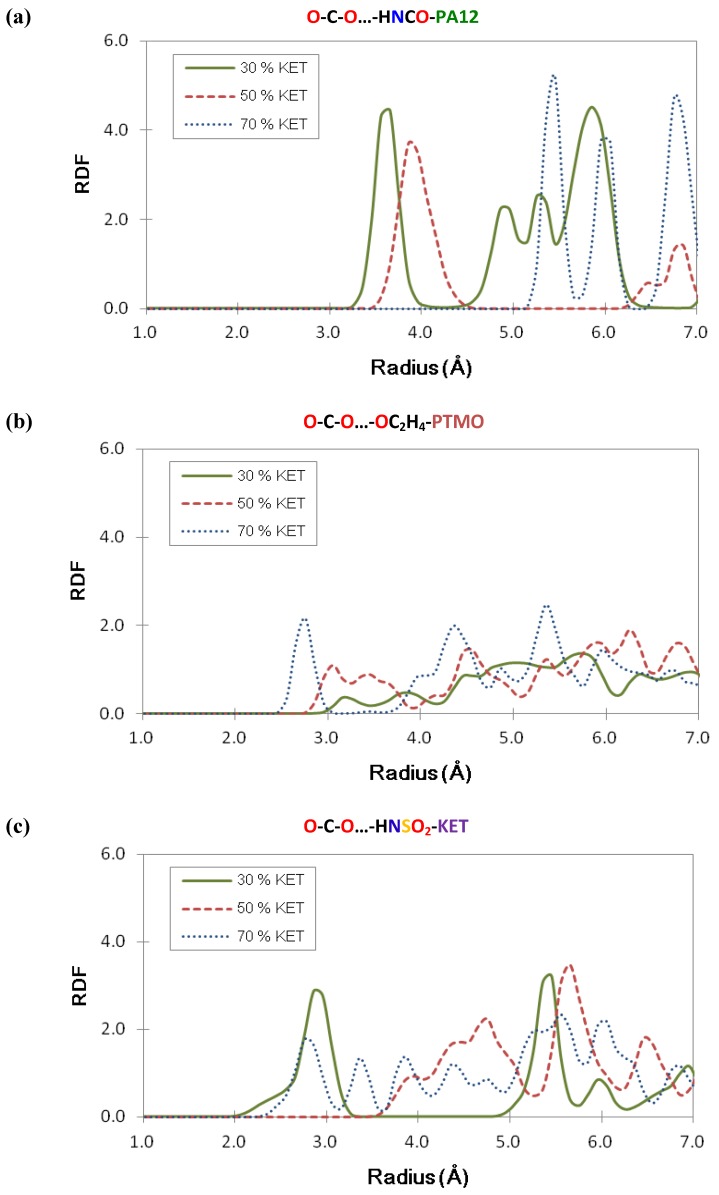
Radial distribution functions (RDF) between the oxygen of CO_2_ and (**a**) the N of PA-12 amidic group; (**b**) the O of the PTMO group; (**c**) the HN of the sulphonamidic group of KET in PEBAX/KET models.

The calculated radial distribution functions, averaged over all atom pairs, are plotted in Figure 8, where in detail, interactions between the oxygen of CO_2_ and the N of the PA-12 amidic group in Figure 8a and the O of the PTMO group in Figure 8b are indicated. Also associations between CO_2_and KET molecules (in detail HN- of sulphonamidic group of KET) were analyzed in PEBAX/KET models.

Generally the peaks observed at distances less than 4 Å are assigned to a specific distance of the closely coupled atoms. At long distances RDF approaches unity, which is quite probable for a purely amorphous system. The peaks in Figure 8a,b indicate that CO_2_ seems to be associated with both the hard PA-12 and with soft PTMO blocks. Correlations are found between carbon dioxide and the amide group of PA-12 for 30% and 50% compositions of PEBAX/KET with first peaks about 3.5 Å and 4 Å while 70% is at about 5.5 Å. More in detail, from the RDF diagrams in Figure 8a, it was found that the first peaks of all membranes are shifted toward the right side while the KET wt % was increased, which suggested that in the highest KET membrane model, the CO_2_ tended to stay far from the PA-12. On the other hand, the interactions between PTMO and CO_2_ are visible, even of slight intensity. The peaks in Figure 8b are shifted to the left side while the KET content is higher, which indicates that in the KET-rich system, there is more CO_2_ that moves toward PTMO. This last indication is analogous to that obtained from pure the PEBAX system [15] where CO_2 _interacts mostly with the ether groups of soft PTMO. In a previous paper [17] the behavior of water was also analyzed: H_2_O prefers to stay near the soft PTMO than to the hard PA-12. 

Figure 8c describes the associations between CO_2_ and KET molecules. The first peaks at about 3 Å for 30% and 70% compositions of PEBAX/KET while the peak of 50% is at about 4.8 Å. RDF indicate that the CO_2_ stays near KET when the loading is 30% and 70%. 

A general remark that could be made is that polar gases and vapor through PEBAX and PEBAX/KET cross the soft PTMO moiety of the co-polymers in the surrounding area of KET clusters mainly when the KET content is higher. KET molecules assemble in clusters of nanometric dimension especially at very high concentrations with polar moieties involved in intermolecular interactions and aromatic ring in a pseudo-stacked orientation (owing to the steric hindrance of the sulphonamide moiety) [17]. 

## 4. Conclusions

In the present work the MD method is applied to a detailed investigation of gas and vapor molecules transport through the complex system of PEBAX modified with different weight percentages of KET. Different theoretical approaches suggest plausible explanations for the gases sorption and diffusion mechanisms that can be controlled according to the final utilization of modified PEBAX membranes. The theoretical data confirms the increase of permeability of all gases and of selectivity polar/non-polar gases in PEBAX/KET membranes with the exception of 70 wt % KET. An inverse relationship between permeability and concentration of KET is outlined: Permeability increases as the additive concentrations decreases. Also the permeability increases from small non-polar gases to larger and polar ones. 

The opposite behavior was noted with regard to the permeability curves observed for solubility: Solubility is directly proportional to the amount of KET in the matrix, with an interesting exception of reduction of solubility when the KET exceeds 50 wt %. This known behavior has also been evidenced by simulations. For diffusion coefficients the theoretical values are much lower than the experimental values, also when small dimension gases are simulated. This is probably due to the rigidity of the mixed matrix system owing to the enlargement of the KET nanocluster size, as shown by the MSD trajectories of the PEBAX chains, but also limitations of the method reduces the readability of results. Interactions between the CO_2_ and PA-12 and PTMO domain of the block copolymer and between CO_2_ and KET molecules were analyzed by radial distribution functions, thus providing useful information about the microscopic structure of assembled modified PEBAX models.
